# Genetic analysis of single disseminated tumor cells in the lymph nodes and bone marrow of patients with head and neck squamous cell carcinoma

**DOI:** 10.1002/1878-0261.13113

**Published:** 2021-10-31

**Authors:** Karl Christoph Sproll, Lara K. Schorn, Benedikt Reising, Sarah Schumacher, Julian Lommen, Norbert R. Kübler, Wolfram Trudo Knoefel, Manfred Beier, Rui P. Neves, Bianca Behrens, Kai Horny, Nikolas H. Stoecklein

**Affiliations:** ^1^ Department of Oral and Maxillofacial Surgery University Hospital Düsseldorf Heinrich‐Heine‐University Düsseldorf Düsseldorf Germany; ^2^ Department of General, Visceral and Pediatric Surgery University Hospital Düsseldorf Heinrich‐Heine‐University Düsseldorf Düsseldorf Germany; ^3^ Institute for Human Genetics University Hospital Düsseldorf Heinrich‐Heine‐University Düsseldorf Düsseldorf Germany; ^4^ Group of Translational Skin Cancer Research (TSCR) University Duisburg‐Essen Essen Germany; ^5^ German Cancer Consortium (DKTK) & German Cancer Research Center (DKFZ) Heidelberg Germany

**Keywords:** bone marrow, disseminated tumor cells, genetic alterations, head and neck squamous cell carcinoma, lymph nodes, minimal residual disease

## Abstract

Considering the limited information on the biology and molecular characteristics of disseminated tumor cells (DTCs) in head and neck squamous cell carcinoma (HNSCC), we examined the genomic alterations in DTCs from HNSCCs and their potential clinical relevance. To analyze both the lymphatic and hematogenous routes of tumor cell dissemination, we investigated samples from lymph nodes (LNs) and bone marrow (BM) of 49 patients using immunofluorescence double staining for epithelial cells expressing cytokeratin 18 (KRT18) and/or epithelial cell adhesion molecules (EpCAM, CD326). The identified marker‐positive cells were isolated by micromanipulation followed by single‐cell whole‐genome amplification and metaphase‐based comparative genomic hybridization (mCGH) to determine genome‐wide copy number alterations. The findings were correlated with clinical parameters and follow‐up data. We detected chromosomal aberrations in KRT18‐ and EpCAM‐positive cells from both compartments; BM‐derived cells showed a significantly higher percentage of aberrant genome (PAG) per cell than cells detected in LNs. No significant association was found between DTC data and clinical follow‐up. Genomic profiling of BM‐DTCs revealed genomic alterations typical for HNSCC, suggesting hematogenous dissemination of subclones around the time of surgery. In contrast, DTC data in LNs revealed that several marker‐positive cells were not of malignant origin, indicating the presence of epithelial glandular inclusions in parts of the processed neck LN samples. Therefore, DTC detection of LNs in the neck based only on epithelial markers is not advisable and requires detection of chromosomal instability (CIN), gene mutations, or additional markers, which have yet to be identified. Nevertheless, our investigation paves the way for larger studies to focus on HNSCC BM‐DTCs with high‐resolution methods to gain deeper insights into the biology of hematogenous metastasis in this cancer.

## Graphical abstract

We isolated disseminated tumor cells from lymph nodes and bone marrow of patients with head and neck squamous cell carcinoma and comprehensively characterized them genetically at the single cell level. While the cells in the lymph nodes are harder to identify, those in the bone marrow showed up as highly aberrant subclones that could possibly become the starting point for distant metastases.

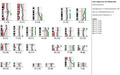

**Abbreviations**
AAO‐HNS: American Academy of Otolaryngology ‐Head and Neck Surgery
BM: bone marrow
BM‐DTCs: bone marrow‐derived DTCs
Chr.: chromosome
CI: confidence interval
CIN: chromosomal instability
CIRC: cytokeratin‐positive interstitial reticulum cells
CNA: copy number alteration
CTC: circulating tumor cell
cTNM: clinical classification of tumor (T), nodes (N), and metastases (M)
DNA: deoxyribonucleic acid
DPBS: Dulbecco's phosphate‐buffered saline
DTC: disseminated tumor cell
EC: esophageal cancer
EGFR: epidermal growth factor receptor
EMT: epithelial‐to‐mesenchymal transition
EpCAM: epithelial cell adhesion molecule
*g*
: acceleration due to gravity g≈9.81m·s^−2^
GO: Gene Ontology
HNSCC: head and neck squamous cell carcinoma
HR: hazard ratio
IF: immunofluorescence
ISCN: International System for Human Cytogenetic Nomenclature
IU: international units
KRT: cytokeratin
LN: lymph node
Mbp: mega base pairs
mCGH: metaphase‐based comparative genomic hybridization
MRD: minimal residual disease
neg: negative
NGS: next‐generation sequencing
PAG: percentage of aberrant genome
PBS: phosphate‐buffered saline
PCR: polymerase chain reaction
pH: potential of hydrogen
pos: positive
pTNM: pathological classification of tumor (T), nodes (N), metastases (M)
TCGA: The Cancer Genome Atlas
UCSC: University of California Santa Cruz
UICC: Union Internationale Contre le Cancer
US: ultrasound
WGA: whole‐genome amplification

## 1 Introduction

Annually, more than 550 000 new cases of malignant tumors are detected in the head and neck region which leads to ~ 300 000 deaths [1]. Head and neck squamous cell carcinoma (HNSCC) accounts for 95% of these cases [2, 3]. In the past 40 years, the generally poor prognosis for HNSCC has barely improved. With recent dramatic improvements in surgical techniques including microvascular reconstruction as the standard of care, precise radiotherapy, targeted anti‐EGFR therapy, and immunotherapy especially with pembrolizumab and nivolumab, long‐term cure seems achievable. For example, in early HNSCC stages without lymph node (LN) metastases, the 5‐year survival rate has increased to more than 80% [4]. However, ~ 20–30% of these patients develop locoregional relapse and even distant metastases at low frequency [5]. These rates are higher in locoregionally advanced stages, without distant metastases [6]. Such relapses seem to emerge from micro‐deposits and individual cancer cells that have disseminated before tumor resection and are termed minimal residual disease (MRD). These MRD cells escape routine diagnostics but can be detected using sensitive molecular detection assays in mesenchymal organs, lymph nodes (LNs), and bone marrow (BM). Immunodetection is commonly used to visualize disseminated tumor cells (DTCs) via epithelial antigens in mesenchymal indicator organs [9]. For HNSCC, the AE‐1/AE‐3 pan‐cytokeratin antibody has been the most widely used for DTC detection, as it recognizes a wide range of acidic and basic cytokeratins [6, 12]. EpCAM (epithelial cell adhesion molecule, CD326) has also been described as a reliable marker, especially for DTCs in LNs [7] or CTCs in blood [8], but has not been used in HNSCC.

Unlike other cancer entities (e.g., breast cancer or gastroesophageal cancer), DTCs are less well studied in HNSCC, but sufficient data have been published to conclude that with some margin, ~ 20–30% of patients harbor epithelial DTCs in the LNs and BM [9]. In addition, in contrast to other cancer entities, the prognostic relevance of DTCs in HNSCC is less well established and tends to be insignificant according to most available studies [9].

To date, the biology or molecular characteristics of DTCs in HNSCC remain unclear. Since the advent of single‐cell analysis, genomic profiling has been performed for DTCs in several cancer entities. For example, genomic DTC profiling in esophageal cancer (EC) revealed that some of the genomic alterations between DTCs from BM and LN diverge and that LN‐DTCs display significantly more aberrations than BM‐DTCs. The latter, in conjunction with findings in BM‐DTCs of breast and prostate cancer patients, was interpreted as a sign of early dissemination into the BM, as the chromosomal copy number alteration (CNA) burden of primary tumors steadily increases during their development and promotes further invasion and metastasis [10]. Interestingly, only highly aberrant DTCs in EC confer a poor prognosis [11].

To gain insight into the genomic makeup of DTCs in HNSCC patients, this study aimed to determine the genomic alterations and to test for differences between DTCs derived from BM and LN as well as their impact on overall and disease‐free survival.

## 2 Materials and methods

### 2.1 Study patients and sample collection

Patients with a primary diagnosis of HNSCC and without previous or simultaneous tumors in another region were included in the study. After routine staging (as reported earlier [12]) and consultation with a multidisciplinary tumor board, they were intended for primary surgical therapy with excision of all tumor‐affected tissues including a clinical safety margin of 10–15 mm, an elective or therapeutic neck dissection, and a defect reconstruction using primary wound closure, local, pedicled regional or free flaps, and of hard tissues by alloplastic reconstruction or free tissue transfer to our clinic for oral and maxillofacial surgery at the University Hospital Düsseldorf. Macroscopically, tumor‐free LNs and BM aspirates were harvested during the surgery. Written informed consent was obtained, and the Ethics Committee of the Medical Faculty of the Heinrich‐Heine‐University Düsseldorf (#3090) approved the study. All procedures performed in studies involving human participants were in accordance with the ethical standards of the institutional and research committee and with the 1964 Helsinki Declaration and its later amendments or comparable ethical standards. BM aspirates (18 mL) were retrieved after a small skin incision from each hip prior to the first incision for tumor surgery into a syringe containing 2 mL of heparin sodium (25 000 IU/5 mL; Ratiopharm^®^, Ulm, Germany; yielding 10 000 IU heparin) and were mixed thoroughly for 2 min. If necessary, the aspirates were stored overnight at 4 °C on a roller incubator. Neck LNs were investigated by ultrasound (US), depicted on a map containing the neck levels according to the American Academy of Otolaryngology‐Head and Neck Surgery (AAO‐HNS), and those most likely to be affected by metastasis but still clinically negative were then identified and harvested during neck dissection [13]. One half of a single LN was retained for assessment, and the second half was sent to the Institute of Pathology at the University Hospital Düsseldorf for routine evaluation. The retained parts of the LNs were again split into halves of which one part was placed into 1× DPBS solution (pH 7.4; Gibco, Invitrogen^®^, Karlsruhe, Germany), and the other part was snap‐frozen in liquid nitrogen.

### 2.2 Single‐cell preparation

Preparation of single‐cell suspensions from BM aspirates and LN samples was performed using a standardized protocol reported earlier [14, 15]. Ten milliliters of BM obtained from the operating room was suspended in 10 mL Hanks salt solution and centrifuged at 170 ***g*** for 10 min. The supernatant was removed, and the pellet was suspended in 20 mL of 1× DPBS buffer (pH 7.4). The cell suspension was then added to 20 mL Ficoll‐Paque (GE Healthcare, Chalfont St. Giles, UK) and centrifuged at 550 ***g*** for 30 min. The interphase containing peripheral blood mononuclear cells (PBMCs) was then removed, resuspended twice with 20 mL 1× DPBS buffer (pH 7.4), and centrifuged at 365 ***g*** for 10 min. The cell pellet was then resuspended in 2 mL of 1× DPBS buffer (pH 7.4) and counted, and the cell concentration was adjusted to 500 000 cells·mL^−1^ in 1× DPBS buffer (pH 7.4). Of these, suspensions, 0.5 mL each (corresponding to 250 000 cells), were placed on an adhesive slide (Menzel^®^, Braunschweig, Germany) containing two fields. After the cell suspension had settled for 30 min, the supernatant was removed, the adhesive slide was dried overnight at room temperature and then stored at −20 °C until the staining procedure.

The LN tissue obtained intraoperatively was freed from the fatty and connective tissue residues and necrotic areas and was cut into ~ 2‐mm^3^ pieces, placed in 1 mL 1× DPBS buffer (pH 7.4) in a Medicon (50 µL, BD Biosciences, San Jose, CA, USA) and, depending on the size of the fragments, the Medimachine (BD Biosciences) was run several times for 60 s. The resulting cell suspension was washed in 10 mL 1× PBS (pH 7.4), centrifuged for 10 min at 200 ***g***, resuspended in 5 mL 1× PBS, filtered through a 70‐µm cell sieve (Greiner Bio‐One, Frickenhausen, Germany), processed, counted, and applied to the adhesive slides similar to the BM.

### 2.3 Double immunofluorescence (IF) staining

For double IF staining of 1 × 10^6^ cells per LN and BM sample, a monoclonal mouse antibody against epithelial cell adhesion molecule (EpCAM; Clone: BerEp4, Dako^®^, Hamburg, Germany) together with a monoclonal rabbit anti‐cytokeratin 18 (CK18; Clone: E431‐1, Abcam^®^, Cambridge, UK) was applied. IF staining was performed using the protocol described by Driemel *et al*. [16]. DTCs were suspected to be among the KRT18‐ and/or EpCAM‐positive non‐granulated round cells with large nuclei located within the same focal plane of the adhesion slide as the peripheral blood lymphocytes to rule out any cross‐contamination.

**Fig. 1:**
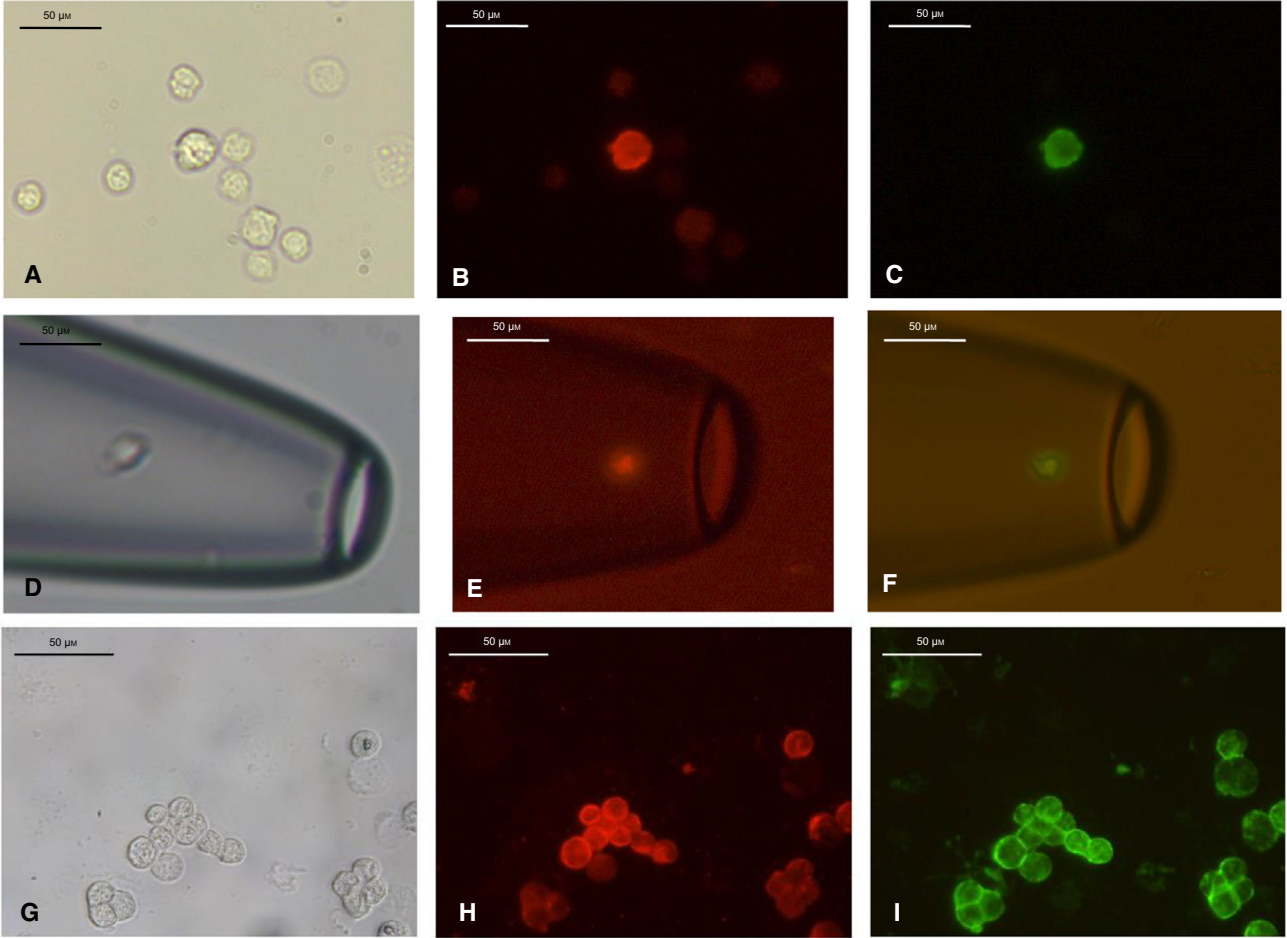
Example of a cytokeratin 18/epithelial cell adhesion molecule (KRT18/EpCAM) double‐positive cell, Pat. #30, LN 4, cell No. T3: (A) brightfield, (B) KRT18 (Cy3, red), and (C) EpCAM (Alexa 488, green). Disseminated tumor cell, detached from the adhesive slide, isolated, and captured in a microhematocrit capillary with the help of a micromanipulator (Eppendorf) at 40× magnification. (D) Bright‐field (from Pat. #49, LN 3, cell No. T3), (E) KRT18 (Cy3, red) and (F) EpCAM (Alexa 488, green). KRT18/EpCAM double immunofluorescence staining of the cell line LN1590 at 40× magnification served as a positive control. (G) Bright‐field, H. KRT18 (Cy3, red), and I. EpCAM (Alexa 488, green). Both epithelial antigens were detected in the control cell line LN1590. Scale bars correspond to 50 µm.

Positively stained cells were isolated using a micromanipulator (Eppendorf^®^, Hamburg, Germany), and contamination with unstained cells was carefully avoided (Fig. 1). Specificity was ensured by double immunostaining a control cell line (LN1590) [14] and the SCC‐4‐cell line [17]), which was positive for both epithelial markers (Fig. 1). Single cells were whole‐genome amplified (WGA) using adapter‐linker/MseI‐PCR as previously described by Klein *et al*. [18], commercialized as the Ampli1 WGA Kit (Silicon Biosystems^®^, Bologna, Italy). To verify the quality of the primary PCR product, a control PCR was performed to detect specific MseI fragments. Two oligonucleotide pairs p53 exon 2/3 (375 bp) and KRT 19 (750 bp) were used for this purpose. Samples were regarded as suitable for mCGH examinations if at least one specific PCR product was successfully amplified.

### 2.4 Metaphase‐based comparative genomic hybridization (mCGH)

mCGH of WGA single‐cell DNA was performed using a standardized protocol as previously reported [11, 19, 20]. The isis software (V 5.5.1; MetaSystems, Altlussheim, Germany) and a fluorescence microscope were used to generate and evaluate mCGH profiles. mCGH karyotypes were labeled according to the International System for Human Cytogenetic Nomenclature (ISCN) [21]. For each sample, the numbers and sites of alterations per cell were determined.

### 2.5 Statistical analysis

Hierarchical cluster analysis was performed with the r statistical Software [22] and the WECCA package for clustering of called aCGH data using default settings (distance measure = “agree”, linkage = “ward”, weight type = “all equal”) [23]. mCGH karyotypes were joined in a composite karyotype for primary HNSCC tumors and LN metastasis, for which more than one sample was analyzed. For each sample, the number of alterations was counted and the mean number of alterations was calculated for BM‐ and LN‐DTCs. The percentage of aberrant genomes per cell (PAG) was calculated to obtain a global measure of chromosomal instability (CIN) as described previously [11]. To this end, the number and size of alterations were considered, each alteration was translated into mega base pairs (Mbp), and the total length of the altered genome (based on hg38 positions) was divided by the total genome size from the UCSC table (3.088269832 Gb) (https://genome.ucsc.edu/cgi‐bin/hgTables?command=start). Alterations at the Chr. 9 and 1 pter‐1p33 were not included in the calculation because of known non‐tumor‐associated imbalances of the mCGH at these loci [24]. A Wilcoxon–Mann–Whitney *U*‐test was performed to compare the percentage of aberrant genomes (PAG) and number of aberrations per cell between the two sample groups. Finally, cells with more than 1% PAG were used as DTCs.

To obtain an approximation of potentially involved genes or pathways, Gene Ontology (GO) term enrichment analysis seemed too speculative because of the large number of genes located in the altered chromosomal regions (Fig. S1). Instead, oncogenes and tumor suppressor genes with a known role in HNSCC were identified. For this purpose, the 328 oncogenes and 82 tumor suppressor genes from the respective gene family of the Molecular Signatures Database v7.4 (https://www.gsea‐msigdb.org/gsea/msigdb/) were used [25]. Of the 328 oncogenes, 54 were selected as relevant for HNSCC based on a PubMed search under the search term "HNSCC AND oncogene" from the years 2001 to 2021 or presence among the 574 most frequently mutated genes in HNSCC in the TCGA database (https://portal.gdc.cancer.gov/exploration?filters; TableS1; the selected tumor suppressor genes are listed in Table S2). Chromosome band locations were extracted from the Ensembl BioMart database and were combined with information regarding the genes on chromosome bands from the UCSC genome table browser, using the reference genome GRCh38. Chromosome regions were matched to genes and gene sets using a custom R script in R version 4.0.5, and plots were created using ggplot2 version 3.3.3 and ggpubr version 0.4.0 [22]. Kaplan–Meier analysis was used to evaluate the influence of the primary tumor size (T‐category), LN status (N‐category), and occurrence of DTCs on disease‐related survival in months for a maximum of 5 years. The endpoint was patient death. Log‐rank tests were used to compare survival data, and Cox regression analysis was used to assess the predictive value of existing DTCs in LNs or BM. We also used Cox regression models to estimate the hazard ratios (HRs) and 95% CIs of the association between DTC detection and death adjusted for T and N stages. Statistical analysis was performed using spss statistics version 21 (SPSS Inc., Chicago, IL, USA). Results with a *P* value < 0.05 were considered significant.

## 3 Results

### 3.1 Detection and isolation of DTCs in HNSCC patients

Overall, 49 patients with HNSCC were included in this study. The locations of the respective primary tumors were as follows: lip mucosa (C00.4) three patients, tongue (C02.0, 1) 12 patients, upper and lower alveolus and gingiva (C03.0, 1) 11 patients, floor of the mouth (C04) 10 patients, hard palate (C05.0, 1) two patients, buccal mucosa (C06.0, 2) seven patients, parotid gland (C07.9), oropharynx (C10.2), nasopharynx (C11.1), and hypopharynx (C13.0) one patient each. The clinical follow‐up interval ranged from 0 to 169 months (average, 60 months; median, 38 months). Table 1 summarizes the remaining relevant clinical data. Even if the patients had LN metastases (cN+, pN+), only LNs that showed no evidence of metastasis in the preoperative clinical examinations (CT, ultrasound) or manual palpation, and dissection in the operating room and were actually tumor‐free on histopathology were selected.

**Table 1:** Staging and grading of HNSCC patients included in this study; the number of patients did not add up to 100% as BM and LN samples were not available from all patients. However, we related the number of positive patients with the number of patients from whom BM and/or LN samples were available.

Patients	*N* = 49	Patients with marker‐positive cells BM	Patients with marker‐positive cells LN
Sex			
Female (Ø73y/a)	19	4/15 (26.7%)	7/16 (43.8%)
Male (Ø64y/a)	30	4/30 (13.33%)	11/24 (45.83%)
pT‐Status			
pT1	7	0/7 (0%)	3/7 (42.86%)
pT2	25	4/21 (19.5%)	11/21 (52.38%)
pT3	12	4/12 (33.3%)	4/4 (100%)
pT4	5	0/5 (0%)	0/4 (0%)
pN‐Status			
pN0	25	4/25 (16%)	12/20 (60%)
pN1‐2	24	4/20 (20%)	6/20 (30%)
M‐Status			
M0	44	7/42 (16.67%)	15/35 (42.86%)
M1	5	1/3 (33.33%)	3/5 (60%)
G‐Status			
G1	1	0/1 (0%)	0/1 (0%)
G2	38	6/37 (16.22%)	16/38 (42.11%)
G3	10	2/7 (28.57%)	2/10 (20%)
R‐Status			
R0	31	4/28 (14.29%)	12/15 (80%)
R1	16	3/15 (20%)	6/13 (46.15%)
R2	2	1/2 (50%)	0/2 (0%)

Altogether, 47 LN preparations and 48 BM samples were available from 40 and 45 patients, respectively. In total, 22 of 47 (46.8%) LNs in 18 of 40 (45%) patients displayed marker‐positive cells. We examined 48 BM aspirates from 45 patients and detected marker‐positive cells in 17.8% (*n* = 8) of the cases. In terms of marker expression, 18 of 47 (38.3%) and 8 of 48 BM samples (16.7%) displayed KRT18^pos^/EpCAM^neg^‐DTCs. KRT18^pos^/EpCAM^pos^ cells were observed in 6.8% (3/47) of the LN samples and 2.1% (1/48) of the BM samples. Only one of the LN samples showed a KRT18^neg^/EpCAM^pos^ cell (2.1%, 1/47). In total, 179 KRT18‐ and/or EpCAM‐positive cells were detected by IF. Further, 113 KRT18^pos^/EpCAM^neg^, 3 KRT18^pos^/EpCAM^pos^, and 3 KRT18^neg^/EpCAM^pos^ cells were found in the LN suspensions, 51 KRT18^pos^/EpCAM^neg^, 5 KRT18^pos^/EpCAM^pos^, and 5 KRT18^neg^/EpCAM^pos^ cells were found in the BM samples (see Fig. S2).

### 3.2 mCGH analyses of single BM‐DTCs and LN‐DTCs

**Fig. 2:**
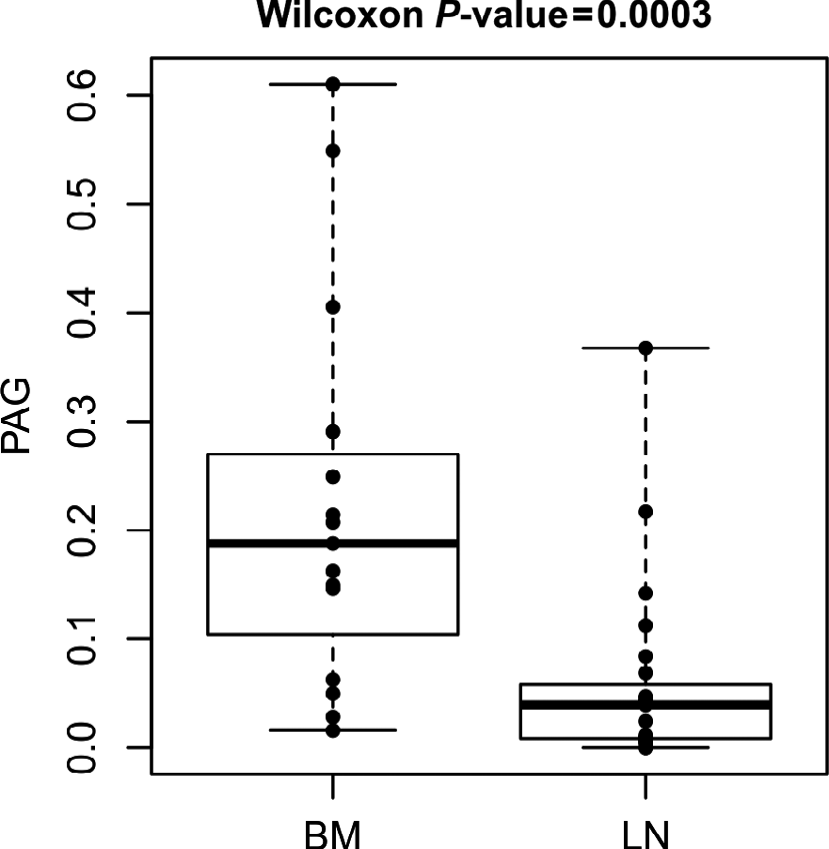
Percentage of aberrant genome per cell (PAG) showing significantly higher values in marker‐positive cells derived from bone marrow (BM) than in those derived from lymph nodes (LN; Wilcoxon–Mann–Whitney *U*‐test: *P* = 0.0003). Box plot with median and interquartile box.

The number of manually micromanipulated cells was limited to three marker‐positive cells per sample. In total, 79 (LN: 52 + BM: 27) cells were isolated via micromanipulation. Of these, 20 cells (LN: 14 + BM: 6) were lost (25.3%; Fig. S2). Therefore, 59 (LN: 38+ BM: 21) cells were successfully isolated as single cells from adhesive slides and transferred to a microcentrifuge tube, accounting for a “pick‐rate” of 59/79 cells (74.7%). After primary amplification, 8/59 (13.6%) of the isolated single cells were positive for one and 32/59 (54.2%) were positive for two specific MseI fragments in the control PCR. Therefore, mCGH could be performed on 40 marker‐positive cells (25 LN‐derived and 15 BM‐derived; Fig. S2). Of these, 38 could be evaluated. In two of the LN‐derived cells, there were no detectable genomic aberrations; therefore, the amplifications and/or deletions in the genomes of 36 cells could be assessed. Amplifications in over 50% of single cells were found on chromosomes 1, 8, 11, 15–17, 19, and 20, those in over 25% of single cells were found on chromosomes 2, 3, 5, 12, 14, and 22, and those in over 10% of cells were found on chromosomes 9 and 10. Deletions were predominantly found on chromosomes 4q, 6q, 9p, 13, and 18 (Table 2). Cumulative mCGH plots of cells from the LN and BM compartments were created and compared (Fig. 2). The mean percentage of aberrations per cell in BM‐DTCs was 15.8%, which was significantly higher than that in LN‐DTCs (5.4%; *P* = 0.0002, Wilcoxon–Mann–Whitney *U*‐test). Accordingly, the PAG of BM‐derived cells was significantly higher than that of LN‐derived cells (*P* = 0.00003, Wilcoxon–Mann–Whitney *U*‐test, Fig. 2). In a previous immunohistochemical study, we found a large number of KRT5/14^pos^ and/or CD44v6^pos^ cells in LNs from pN0 HNSCC patients that did not show the morphological criteria of tumor cells but those of glandular cells, whereas some had a tubular arrangement or that of reticulum cells; we thus classified cells with no or only very low CIN (PAG ≤ 1%) as non‐neoplastic cells. This affected 9 of 23 marker‐positive LN‐derived cells. All 15 BM‐derived cells, except for one (#25, cell T4: 0.69% PAG) had a PAG of more than 1% [12]. Overall, we were able to detect LN‐DTCs in nine patients (7: pN0, 1: pN2a, 1: pN2b) and BM‐DTCs in five patients (4: cM0, 1: pM1). Thus, there was no correlation with a higher N‐ or M‐status of the patients.

**Table 2:** Locations of amplifications and deletions in bone marrow‐ or lymph node‐derived disseminated tumor cells (BM‐ and LN‐DTCs) determined in a relevant number of cases. Locations are linked to the genes and products that may be involved. The GISTIC module identifies regions of the genome that are significantly amplified or deleted across a set of samples (https://www.genepattern.org/modules/docs/GISTIC_2.0).

	BM‐DTC (%)	LN‐DTC (%)	HNSCC GISTIC *Q*‐values [33]	Gene involved/affected product
Location of amplification				
11q13	60	4.3	0	*CCND1* (Cyclin D1) *FGF 3* and *FGF 4* *FADD*
8q24	53.3	2.3	5.3246e‐52	*MYC* (C‐myc) *POU5F1B* (Oct‐4)
3q26	46.7	8.7	5.5054e‐119	*PIK3CA (*PI3K‐AKT signaling cascade)
17q22	33.3	8.7	NA	*RAD51C*
9q34	33.3	8.7	9.73e‐18	*NOTCH 1*
5p15	9.1	8	2.092e‐53	*PDCD6* (programmed cell death protein 6) *CEP72* (Centrosomal Protein 72)
Location of deletion				
4q35.2	40	4.3	3.5081e‐66	*FAT 1*
18q12qter	27.2	16	1.8465e‐65	*ADNP2* (Activity‐dependent neuroprotector homeobox) *PARD6G* (Par‐6 Family Cell Polarity Regulator Gamma)
9p21	26.7	4.3	7.4273e‐160	*CDKN2A* (cyclin‐dependent kinase inhibitor 2A, p16/INK4A)
13q12q14, 13q21q22 13q31q32	26.7	4.3	7.6449e‐16	*BRCA2, RB1* (Dysplasia into CIS (carcinoma *in situ*))

Finally, 14 DTCs per compartment were used for further analyses.

**Fig. 3:**
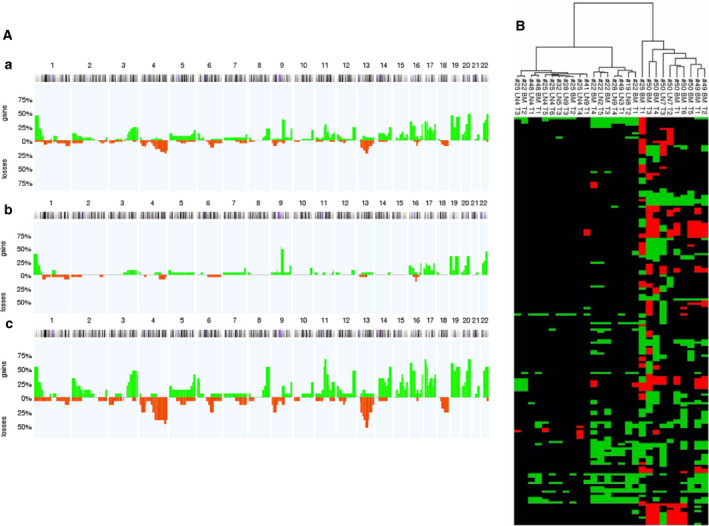
(A) Metaphase‐based comparative genomic hybridization (mCGH) analysis of marker‐positive cells showing genomic gains and losses allocated to chromosomes. Cumulative mCGH plot of (a) all analyzed marker‐positive cells from bone marrow (BM) and lymph node (LN) samples, (b) 23 LN‐derived marker‐positive cells, and (c) 15 BM‐derived marker‐positive cells. Horizontal axis = chromosome number, vertical axis = percentage of genomic aberrations, green = amplifications and red = deletions. (B) Dendrogram of similarity analyses of all disseminated tumor cells (DTCs; percentage of aberrant genome (PAG) > 1%) from bone marrow (BM) and lymph nodes (LNs) using r software. In the dendrogram, the chromosomes are in the ascending order on the *y*‐axis from top to bottom (no visual numbering). The respective DTC is shown on the *x*‐axis. The dendrogram is on the top of the *x*‐axis. Green boxes indicate amplifications and red box indicates deletions. The first sample number (#) corresponds to the patient number; LN = LNs with the corresponding numbering; BM = BM with the corresponding numbering T = tumor cell with the corresponding numbering.

To distinguish whether BM‐derived cells showed more aberrations than LN‐derived cells because of their origin (LN vs. BM) or because of their expression status (KRT18^pos^/EpCAM^neg^, KRT18^pos^/EpCAM^pos^, and KRT18^neg^/EpCAM^pos^), the number of aberrations and PAG was correlated with the expression status. However, there were no significant differences in the number of aberrations and PAG between the three groups. Considering the small number of KRT18^neg^/EpCAM^pos^ cells (only one cell), these cells could not be evaluated. Further, we evaluated whether this difference would also occur in a patient‐specific manner. Marker‐positive cells and DTCs of BM (23 marker‐positive cells/14 DTCs) and LN (15 marker‐positive cells/9 DTCs) of the same five patients (# 22, 25, 48, 49, and 50) could be isolated and evaluated by mCGH, also showing significantly more genomic aberrations in BM‐DTCs than in LN‐DTCs (*P* = 0.0105, Wilcoxon–Mann–Whitney *U*‐test; Fig. S3A,B). Hierarchical analyses were carried out using the r software (R Core Team, Vienna, Austria), which determined the clonal relationship of BM‐ and LN‐DTCs. The similarities of genomic changes were shown by their close proximity in the dendrogram (Fig. 3) and indicated that DTCs were grouped roughly according to their origins, that is, LN and BM. DTCs from BM and LN of the same patient also showed a clonal relationship (pointing to an intratumoral homogeneity) and tended to have a stronger relationship as compared to DTCs from different patients (intertumoral heterogeneity). Of the five patients for whom DTCs were available from both compartments, the cells of patient # 22 (UICC II) partially clustered together, those of patients # 25 (UICC II) and 49 (UICC III) were found in different clusters and those of patients # 48 (UICC IV) and 50 (UICC III) in the immediate vicinity (see Fig. 3).

**Fig. 4:**
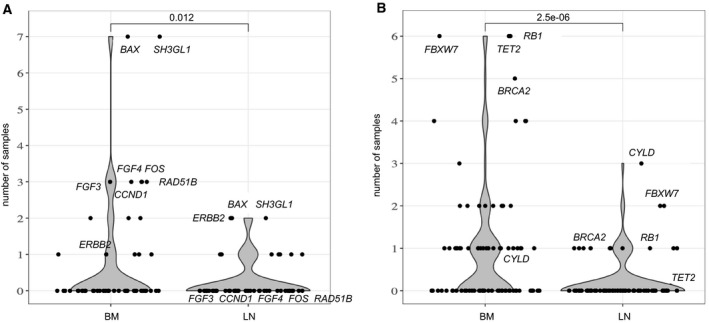
Number of disseminated tumor cells with copy number alterations in chromosomal regions where known oncogenes and tumor suppressor genes for head and neck squamous cell carcinoma (HNSCC) are located. (A) Number of bone marrow‐derived (BM) and lymph node‐derived (LN) cells (*y*‐axis) with amplifications in HNSCC oncogenes (A) or losses in tumor suppressors (B) (Tables 3 and 4). Each point corresponds to a gene locus and the most frequently amplified, and deleted gene loci are labeled. *P*‐values are calculated using a paired Wilcoxon rank‐sum test.

A comparison of affected genes within the chromosomal segments showed that BM‐DTCs have more amplifications in HNSCC‐relevant oncogenes Table 3, *P* = 0.012; paired Wilcoxon rank‐sum test) and deletions in tumor suppressor genes (Table 4, *P *= 2.e‐06; paired Wilcoxon rank‐sum test) compared to LN‐DTCs (Fig. 4). For example, the most frequently affected oncogenes *BAX* and *SH3GL1* were amplified in 7/14 (50%) BM‐DTCs, but only in 2/14 (14%) LN‐DTCs. The tumor suppressor genes *RB1* and *TET2* were lost in 6/14 (42.8%) BM‐DTCs, whereas only one LN‐DTC showed *RB1* loss, and no LN‐DTC showed *TET2* loss.

**Table 3:** List of oncogenes potentially affected due to their position on an amplified chromosome segment per sample (cell). LK stands for LN, and KM stands for BM. The nomenclature of the individual cells is for example: # 19 LK8 T2: patient 19, lymph node 8, tumor cell 2. Each gene represents a point in Fig. 4A.

sample ID	HNSCC oncogenes on amplified region
#19 LK8 T2	SH3GL1, BAX
#22 LK2 T5	CCND3, ERBB2
#25 LK4 T3	
#25 LK4 T4	
#25 LK4 T5	
#25 LK4 T6	
#28 LK9 T3	
#28 LK9 T4	
#32 LK5 T3	
#41 LK9 T1	
#48 LK4 T1	SH3GL1, BAX
#49 LK3 T1	
#50 LK7 T2	CREB3L2, MET
#50 LK7 T3	NFIB, JAK2, GNAQ, NOTCH1, ERBB2
#22 KM T1	NRAS, ERBB2, SH3GL1, BAX
#22 KM T2	
#22 KM T3	SH3GL1, BAX
#22 KM T4	CCND3, FGF3, CCND1, FGF4, SH3GL1, BAX
#25 KM T1	HRAS, FGF3, CCND1, FGF4, SH3GL1
#25 KM T2	
#48 KM T1	SH3GL1, BAX
#49 KM T1	FGF3, CCND1, FGF4, SH3GL1, BAX
#49 KM T2	SH3GL1, BAX
#50 KM T1	FOS, RAD51B
#50 KM T3	MDM2, FOS, RAD51B
#50 KM T4	KDM5A, CCND2, KRAS, FOS, RAD51B
#50 KM T5	KDM5A, CCND2, KRAS, BAX
#50 KM T6	

**Table 4:** List of tumor suppressor genes potentially affected due to their position on a lost chromosome segment per sample (cell). LK stands for LN, and KM stands for BM. The nomenclature of the individual cells is for example: # 19 LK8 T2: patient 19, lymph node 8, tumor cell 2. Each gene represents a point in Fig. 4B.

Sample ID	Tumor suppressor genes on regions with chromosomal losses
#19 LK8 T2	
#22 LK2 T5	
#25 LK4 T3	CYLD
#25 LK4 T4	CYLD, CDH1
#25 LK4 T5	CYLD
#25 LK4 T6	
#28 LK9 T3	
#28 LK9 T4	
#32 LK5 T3	
#41 LK9 T1	FBXW7
#48 LK4 T1	
#49 LK3 T1	
#50 LK7 T2	CDC73, SDHC, FH, PHOX2B, FBXW7, KDM5C, KDM6A
#50 LK7 T3	FH, MSH2, MSH6, TNFAIP3, RB1, BRCA2
#22 KM T1	
#22 KM T2	
#22 KM T3	
#22 KM T4	RB1
#25 KM T1	SDHB, CDKN2C, MUTYH, CDC73, SDHC, FH, MSH2, MSH6, PMS1, APC, PIK3R1, GATA3, KLF6, ERCC5, EP300
#25 KM T2	
#48 KM T1	
#49 KM T1	PHOX2B, FBXW7, TET2, CDKN2A, RB1, BRCA2, SMAD4
#49 KM T2	FBXW7, TET2, CDKN2A, FANCG, RB1, BRCA2, SMAD4
#50 KM T1	FBXW7, TET2, RB1, BRCA2, AMER1, KDM5C, KDM6A
#50 KM T3	MLH1, VHL, XPC, FANCD2, SETD2, PHOX2B, FBXW7, TET2, TNFAIP3, CDKN2A, FANCG, FANCF, WT1, DDB2, EXT2, SDHD, ATM, RB1, BRCA2, ERCC5, SMAD4, AMER1, KDM5C, KDM6A
#50 KM T4	FH, VHL, XPC, FANCD2, FBXW7, TET2, WRN, RB1, BRCA2, CYLD, AMER1, KDM5C, KDM6A
#50 KM T5	FBXW7, TET2, APC, CDKN2A
#50 KM T6	SMAD4, KDM5C, KDM6A

### 3.3 Prognostic significance of DTC detection

Forty‐nine patients were included in the analysis, and disease‐related survival was evaluated in months for a period of at least 10 years. Significant differences were detected using log‐rank tests. Disease‐related survival dropped significantly (*P* = 0.0054, log‐rank test) with tumor size. The median survival rates decreased with tumor stage. Patients with LN metastasis (N1‐3) showed shorter survival rates than those in patients without LN metastasis (*P* = 0.012, log‐rank test, Fig. S4). For survival analysis in correlation with marker‐positive cells, patients with at least one KRT18^pos^ and/or EpCAM^pos^ cell in the BM or LNs were compared with patients without the detection of these cells. The median survival of patients with marker‐positive cells was longer (80.4 months ± 16.8) than that of patients without marker‐positive cells (48.4 months ± 9), but this difference was not statistically significant (*P* = 0.220, log‐rank test). Patients harboring marker‐positive cells in the BM had a shorter disease‐related survival (52.9 months ± 19.9) compared to that in patients without marker‐positive cells in the BM (64.4 months ± 10.3, *P* = 0.64, log‐rank test) whereas patients with positive cells in LNs seemed to live longer (90.9 months ± 18.5) than patients without marker‐positive cells found in the LNs (45.9 months ± 8.5, *P* = 0.13, log‐rank test) (Fig. S4). In the multivariable analysis using multiple Cox regressions, the pT and pN stages were also included in addition to the detection of marker‐positive cells. Detection of marker‐positive cells did not influence patient survival with respect to tumor size (*P* = 0.344, Cox regression), LN status (*P* = 0.449, Cox regression), or both (*P* = 0.474, Cox regression). Furthermore, for the maximum number of aberrations in the marker‐positive cells from the BM (*P* = 0.281, Cox regression), the LNs (*P* = 0.386, Cox regression), or their combination (*P* = 0.154, Cox regression), a significant association with 5‐year survival could not be established. Even if only the DTCs are considered (PAG > 1%), there is no significant correlation with the 5‐year survival for the detection of BM‐DTCs (*P* = 0.508; Cox regression), LN‐DTCs (*P* = 0.099; Cox regression) or the detection of DTCs from both compartments (*P* = 0.118, Cox regression).

## 4 Discussion

In this study, we evaluated the genome‐wide CNAs of DTCs derived from BM and LN samples of HNSCC patients via micromanipulation using a low‐resolution method. We found that half of the DTCs showed alterations typical of primary HNSCC. As observed in previous genomic DTC profiling studies, several DTCs harbored no or very few CNAs. However, this was a typical finding in BM samples from patients with non‐metastatic cancer, whereas DTCs isolated from LN samples showed a genomic profile expected from the tumor entity and stage, with a significantly higher number of alterations compared to BM‐DTCs [19]. As we used the same experimental workflow for our study, including the anti‐KRT18/EpCAM‐immunodetection assay as previously published [11, 16], the observation of very few alterations in our LN‐DTCs was rather surprising. However, to isolate marker‐positive DTCs from LNs, they were mechanically disintegrated to generate single‐cell suspensions. The cell suspensions were then sedimented on adhesive slides for subsequent immunodetection and isolation. We analyzed samples from 49 patients with primary HNSCC; While most DTCs in our study were KRT18^pos^/EpCAM^neg^ (*n* = 164), only eight DTCs displayed the KRT18^pos^/EpCAM^pos^ or KRT18^neg^/EpCAM^pos^ (*n* = 8) phenotype. Notably, 94.9% of LN‐DTCs were negative for EpCAM. This is in contrast to previous studies on esophageal cancer [7], in which most LN‐DTCs were EpCAM‐positive. This was interpreted as a sign of an active and proliferating phenotype in these DTCs, as corroborated by *in vitro* data, and its association with poor survival [11]. There are several potential explanations for the observed low EpCAM expression in HNSCC‐DTCs. Similar to esophageal cancer DTCs, environmental cues from the BM may drive cancer cells in a non‐proliferative quiescent/dormant state, which agrees with an EpCAM‐poor/negative phenotype [9]. Furthermore, the low detection frequency of circulating tumor cells (CTCs) in HNSCC with EpCAM‐based methods also indicates low or absent EpCAM expression in DTCs. Interestingly, the CTC detection frequency in HNSCC can be increased by more than 300% when an EpCAM‐independent method is applied in a side‐by‐side comparison with the EpCAM‐based CellSearch system [26]. Considering that EpCAM positivity in primary HNSCCs is correlated with good prognosis and epithelial differentiation [27], it is conceivable that isolated HNSCC‐DTCs tend to be EpCAM‐low or EpCAM‐negative, for example, triggered by epithelial‐to‐mesenchymal transition (EMT) [28].

However, in the present study on HNSCC, no correlation was observed with clinical follow‐up data beyond a non‐significant trend toward reduced survival in BM‐DTC‐positive patients, which is in contrast with data from other studies on HNSCC [9, 29].

At first glance, the low aberration of epithelial marker‐positive LN cells appears puzzling. A simple explanation could be that these cells are ectopic KRT‐positive salivary gland derivatives, which embryologically develop together with neck LNs and can be inclusions of glandular tissue in LNs [12]. In tissue slides, irregularly stained non‐malignant epithelial marker‐positive cells can be identified to some extent by their morphology [12] and excluded from further analysis. In our study, these cells posed a problem for our approach when working with LN suspensions without the morphologic context of histological tissue sections. Most likely, KRT‐positive cells with very few small alterations (maximum > 0–1% PAG; *n* = 7) or no alteration at all (*n* = 2) were normal epithelial cells derived from such inclusions. The very few mCGH alterations are most likely noise, which despite all control experiments [24], is not untypical for this method. Furthermore, mesenchymal cells such as fibroblastic reticulum cells (CK‐positive interstitial reticulum cells, CIRCs) can also express KRT18 in reactive LNs [30] and occur in large numbers in tumor‐draining LNs that are subcapsular in the paracortical regions [31, 32]. However, 11 of the 14 LN‐DTCs displayed typical HNSCC copy number alterations similar to BM‐DTCs [33]. In addition, we saw in the five patients with available material from both compartments that the LN‐DTCs sometimes cluster very closely with the BM‐DTCs of the same patient. Occurring in 53.3% of BM‐DTCs, the most frequently observed alterations were gains on chromosome 8q24 containing the *MYC* gene coding for the transcription factor c‐myc, which is in accordance with the current literature [34]. C‐myc regulates ~ 15% of human genes and induces gene expression. Aberrant *c‐myc* expression may result in uncontrolled gene expression, even in protooncogenes [35]. C‐Myc belongs to the mitogenic signaling pathway downstream of EGFR and may contribute to the limited clinical effectiveness of EGFR inhibitors despite frequent EGFR‐overexpression in HNSCC [36]. Aberrations in 8q24 have also been associated with poor prognosis in patients with other malignancies, such as breast cancer [37]. In the present study, gains on chromosome 11q13 were detected in 28.9% of LN‐DTCS and BM‐DTCs. This region includes the *CCND1* gene that encodes cyclin D1, which can be found in 30–60% of HNSCC cases [38, 39]. *CCND1* gains and *CDKN2A* loss constitute two of the most common genomic alterations in HNSCC and facilitate cell cycle progression and cell survival [40]. Amplifications on chromosome 3 were observed in 46.7% of BM‐DTCs and only in 8.7% of LN‐DTCs. Speicher *et al*. [41] observed amplifications primarily on 3q26qter in primary HNSCC tumors. Genes located at 3q26 are involved in the PI3K‐AKT‐signaling pathway and play a role in regulating cell growth, proliferation, and motility. Kozaki *et al*. [42] identified amplifications in subunit alpha of the *PIK3CA‐gene* encoding phosphatidylinositol‐4,5‐bisphosphate‐3‐kinase in HNSCC patients. Gains on 3q26 in primary HNSCC tissues are associated with the final stages of invasive carcinoma [43]. Further amplifications were found on chromosome 17q22. This region harbors *RAD51C,* which plays a significant role in DNA double‐strand repair [44]. Scheckenbach *et al*. [45] recently showed that amplifications in *RAD51C* represent a genetic risk profile for HNSCC. Further frequent deletions on BM‐DTCs targeted 9p21 that harbors the locus of *CDKN2A* (p16/INK4A), encoding p16, which inhibits cyclin‐dependent kinases such as CDK4 and CDK6 (*cyclin‐dependent kinase 4 and 6*). They phosphorylate retinoblastoma protein (pRb), leading to uncontrolled switching of the cell cycle from the G1‐phase to the S phase and uncontrolled DNA replication [46]. Cyclin D1 activates CDK4 and CDK6. These findings demonstrate the complexity and interaction of amplifications in the chromosome region 11q3 (activating cyclin D1) and deletion of chromosome region 9p21 (inactivation of p16), which can lead to uncontrolled proliferation and tumor progression [47]. In turn, losses at 9p21 are mainly found in the stage of tumor development, which marks the transition from normal mucosa to benign squamous hyperplasia or an alternate precursor lesion [48].

The relevance of comparing the number of DTCs with altered oncogenes and tumor suppressors is limited, as we only observed large chromosomal rearrangements and missed smaller, local amplifications or deletions. Furthermore, our data warrant careful interpretation because of the small number of cases in this study and the low resolution of mCGH used here. Future studies will need to apply modern NGS‐based technologies for genomic profiling; further, additional markers are needed to better identify LN‐DTCs in HNSCC. Clearly, our study demonstrates that this will be a challenging task because immunodetection in LNs commonly relies on the epithelial phenotype, which can be misleading in neck LNs and needs to be carefully considered before applying expensive modern genomic technologies.

## 5 Conclusions

Taken together, we gained insight into the genomic characteristics of DTCs isolated from BM and LN samples. On the one hand, our study revealed that reliable detection of DTCs in cell suspension is impossible with the established marker‐based detection systems because of irregular glandular tissue enclosed in neck LNs. On the other hand, BM‐DTCs displayed aberrations in the expected range typical for HNSCC, demonstrating early hematogenous dissemination of aberrant subclones to distant sites, which might have the proclivity to form metastases. Thus, future studies with larger patient cohorts should focus on characterizing these BM‐DTCs to identify vulnerabilities enabling better prevention of metastasis, which is an increasing clinical problem in patients with HNSCC after successful multimodal local treatment.

## Conflict of interest

The authors declare no conflict of interest.

## Author contributions

CS provided the BM and LN samples and clinical follow‐up data; BR, SS, RN, and BB conducted the experiments and collected the data; and LS, JL, MB, and KH analyzed the data. LS, CS, and NHS wrote the manuscript; NRK, WTK, and KH critically revised the paper. NHS and CS initiated the research, supervised experiments, and had the idea of publishing this paper.

## Supporting information


**Fig. S1**. Overview of the number of genes present in the respective chromosomal segment with amplifications (A) or losses (B) of each of the 48 disseminated tumor cells. Each point corresponds to an amplified (A) or deleted (B) chromosomal region containing the indicated genes on the y‐axis. The nomenclature of the individual cells is for example: # 22 KM T1: patient 22, cell from the bone marrow (KM), tumor cell 1. Gray box plots with median, interquartile range (IQR)), and whiskers with a maximum IQR of 1.5.

**Fig. S2**. Classification and enumeration of the cells according to the marker constellation (cytokeratin 18 (KRT18)^pos^/epithelial cell adhesion molecule (EpCAM)^neg^, KRT18^pos^/EpCAM^pos^ and KRT18^neg^/EpCAM^pos^) and stage in the protocol (visual screening of the staining, successful isolation by micromanipulation and successful amplification). Blue bars: lymph node‐derived cells (LN), orange bars: bone marrow‐derived cells (BM).

**Fig. S3**. A. Cumulative mCGH plots of marker‐positive cells of five patients for whom both, lymph node (LN)‐ and bone marrow (BM)‐samples were available. a. 12 cytokeratin 18/epithelial cell adhesion molecule (KRT18^pos^/EpCAM^neg^) cells from LN samples and b. 15 KRT18^pos^/EpCAM^neg^ and KRT18^pos^/EpCAM^pos^ cells from the BM samples of the five patients # 22, 25, 48, 49, and 50. Horizontal axis = chromosome number; vertical axis = percentage of genomic aberrations; green = amplification and red = deletion. B. Number of aberrations (y‐axis) in marker‐positive cells of the same five patients (# 22, 25, 48, 49, and 50) with both LN and BM samples (x‐axis) in a dot plot diagram. BM‐derived cells displayed significantly (Mann–Whitney U‐test: p = 0.0105) more genomic aberrations than LN‐derived cells. A black dot stands for a LN‐derived cell, a gray one for a BM‐derived cell. The spread is indicated by the lower and upper crossbars. The mean value by the middle crossbar.

**Fig. S4**. Disease‐related survival depending on a: tumor size and b: lymph node (LN) metastasis (pN‐status), detected disseminated tumor cells (DTCs) in c: bone marrow (BM), d: DTCs in LNs, and e: DTCs in BM or LNs of head and neck squamous cell carcinoma patients.

**Table S1**. List of the identified head and neck squamous cell carcinoma (HNSCC)‐relevant 54 oncogenes that were used to search for potentially relevant genes on altered chromosome sections of the DTCs.

**Table S2**. List of the identified head and neck squamous cell carcinoma (HNSCC)‐relevant 82 tumor suppressor genes that were used to search for potentially relevant genes on altered chromosome sections of the DTCs.

## Data Availability

Data will be made available by the corresponding author upon reasonable request.
