# Supplementary figures and images for: Genetic analysis of single disseminated tumor cells in the lymph nodes and bone marrow of patients with head and neck squamous cell carcinoma
Source: Mol Oncol. 2021 Oct 31;16(2):333–46. doi: 10.1002/1878-0261.13113 (PMC8763651; doi:10.1002/1878-0261.13113)

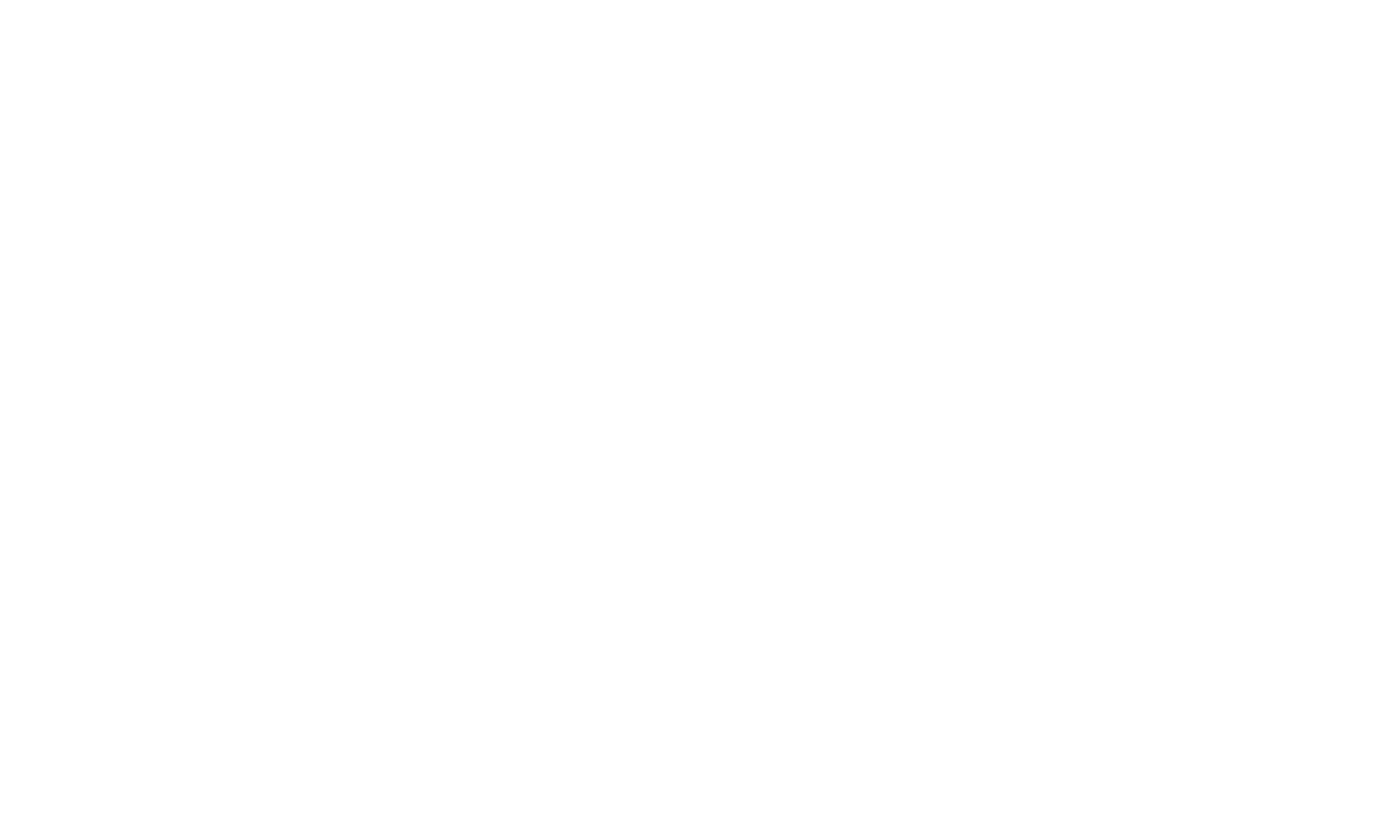

Supplement: Supplementary file 1 — Fig. S1. Overview of the number of genes present in the respective chromosomal segment with amplifications (A) or losses (B) of each of the 48 disseminated tumor cells. Each point corresponds to an amplified (A) or deleted (B) chromosomal region containing the indicated genes on the y‐axis. The nomenclature of the individual cells is for example: # 22 KM T1: patient 22, cell from the bone marrow (KM), tumor cell 1. Gray box plots with median, interquartile range (IQR)), and whiskers with a maximum IQR of 1.5. [file MOL2-16-333-s003.tif]

## Slide 1
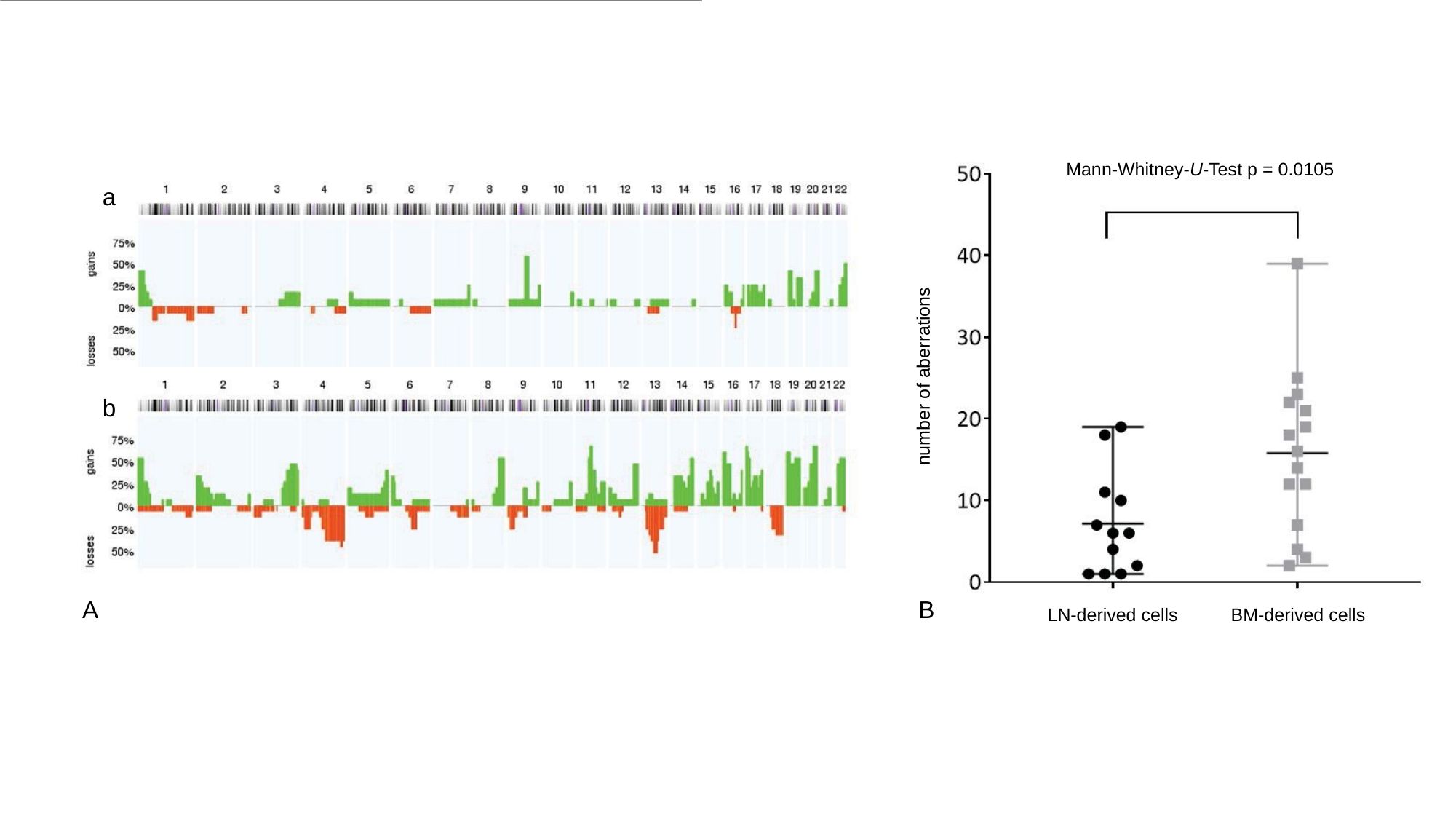

Mann-Whitney-U-Test p = 0.0105
a
number of aberrations
b
A
B
BM-derived cells
LN-derived cells

Supplement: Supplementary file 3 — Fig. S3. A. Cumulative mCGH plots of marker‐positive cells of five patients for whom both, lymph node (LN)‐ and bone marrow (BM)‐samples were available. a. 12 cytokeratin 18/epithelial cell adhesion molecule (KRT18pos/EpCAMneg) cells from LN samples and b. 15 KRT18pos/EpCAMneg and KRT18pos/EpCAMpos cells from the BM samples of the five patients # 22, 25, 48, 49, and 50. Horizontal axis = chromosome number; vertical axis = percentage of genomic aberrations; green = amplification and red = deletion. B. Number of aberrations (y‐axis) in marker‐positive cells of the same five patients (# 22, 25, 48, 49, and 50) with both LN and BM samples (x‐axis) in a dot plot diagram. BM‐derived cells displayed significantly (Mann–Whitney U‐test: p = 0.0105) more genomic aberrations than LN‐derived cells. A black dot stands for a LN‐derived cell, a gray one for a BM‐derived cell. The spread is indicated by the lower and upper crossbars. The mean value by the middle crossbar. [file MOL2-16-333-s001.pptx]

## Slide 1
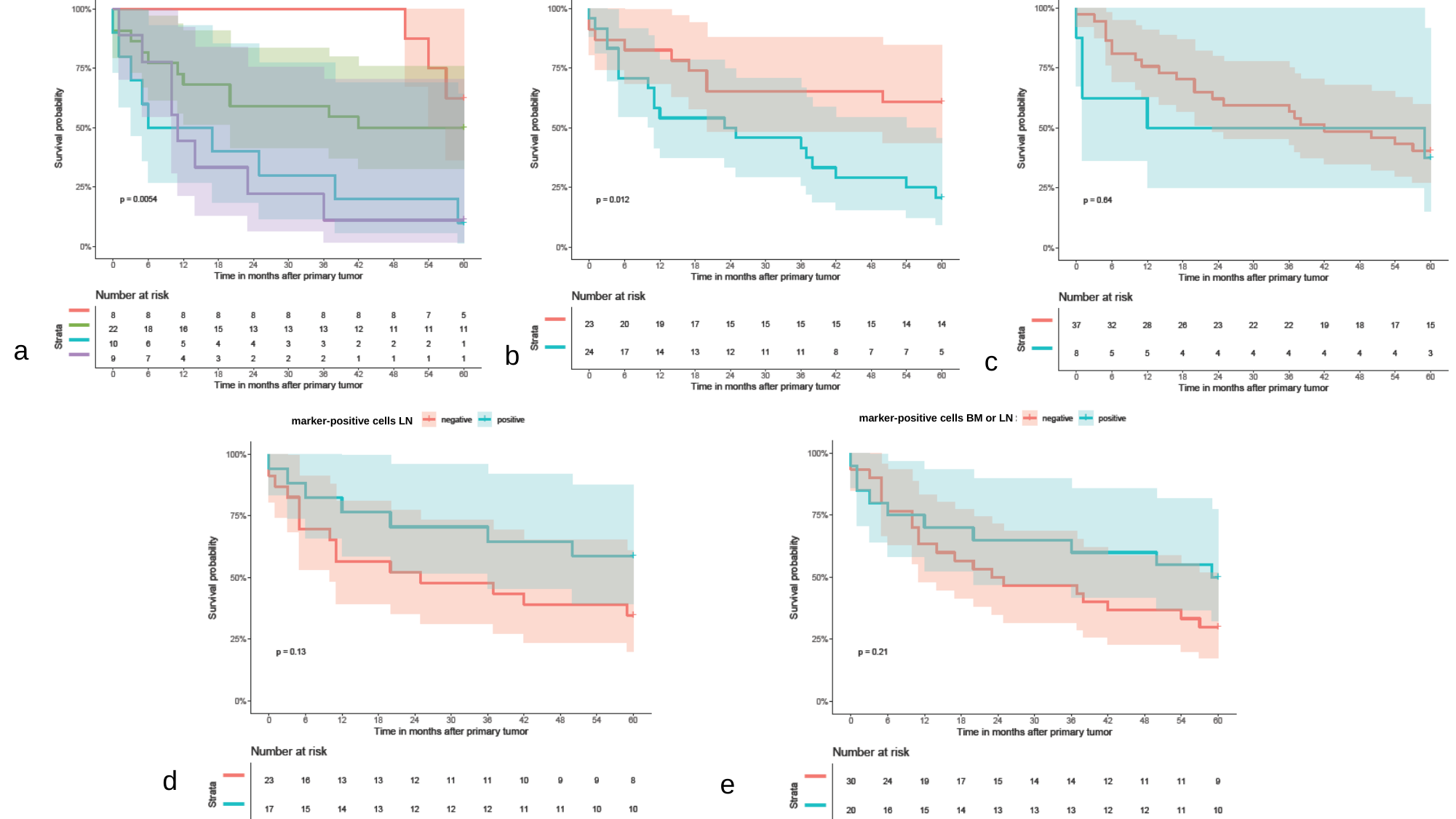

marker-positive cells BM
a
b
c
marker-positive cells BM or LN
marker-positive cells LN
d
e

Supplement: Supplementary file 4 — Fig. S4. Disease‐related survival depending on a: tumor size and b: lymph node (LN) metastasis (pN‐status), detected disseminated tumor cells (DTCs) in c: bone marrow (BM), d: DTCs in LNs, and e: DTCs in BM or LNs of head and neck squamous cell carcinoma patients. [file MOL2-16-333-s002.pptx]
