# Supplementary material for: Genetic analysis of single disseminated tumor cells in the lymph nodes and bone marrow of patients with head and neck squamous cell carcinoma
Source: Mol Oncol. 2021 Oct 31;16(2):333–46. doi: 10.1002/1878-0261.13113 (PMC8763651; doi:10.1002/1878-0261.13113)
Supplement: Supplementary file 2 — Fig. S2. Classification and enumeration of the cells according to the marker constellation (cytokeratin 18 (KRT18)pos/epithelial cell adhesion molecule (EpCAM)neg, KRT18pos/EpCAMpos and KRT18neg/EpCAMpos) and stage in the protocol (visual screening of the staining, successful isolation by micromanipulation and successful amplification). Blue bars: lymph node‐derived cells (LN), orange bars: bone marrow‐derived cells (BM). [file MOL2-16-333-s006.pptx]

## Slide 1
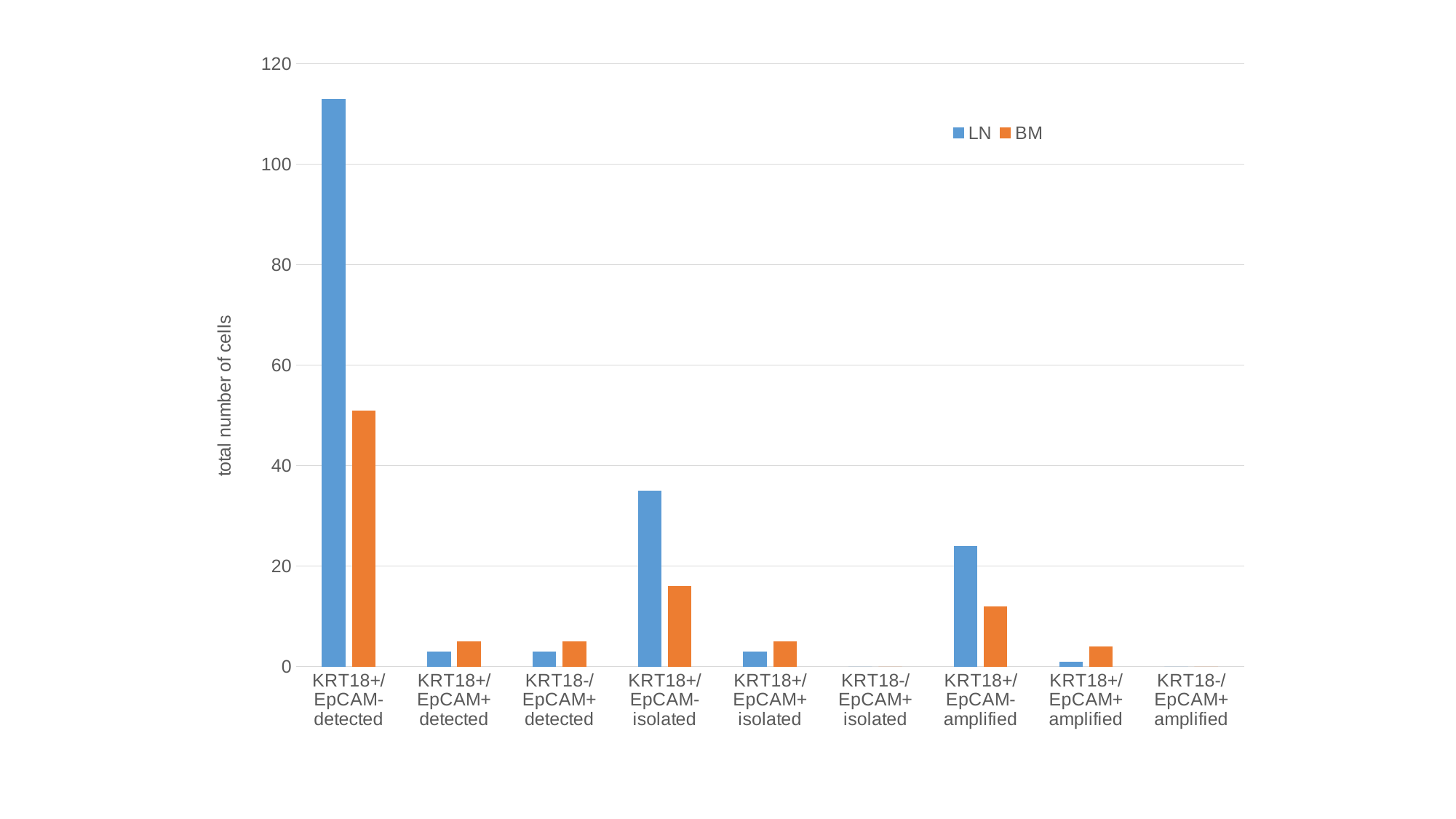

### Chart
| Category | LN | BM |
|---|---|---|
| KRT18+/EpCAM- detected | 113.0 | 51.0 |
| KRT18+/EpCAM+ detected | 3.0 | 5.0 |
| KRT18-/EpCAM+ detected | 3.0 | 5.0 |
| KRT18+/EpCAM- isolated | 35.0 | 16.0 |
| KRT18+/EpCAM+ isolated | 3.0 | 5.0 |
| KRT18-/EpCAM+ isolated | 0.0 | 0.0 |
| KRT18+/EpCAM- amplified | 24.0 | 12.0 |
| KRT18+/EpCAM+ amplified | 1.0 | 4.0 |
| KRT18-/EpCAM+ amplified | 0.0 | 0.0 |
